# Machine Learning Models for Survival and Neurological Outcome Prediction of Out-of-Hospital Cardiac Arrest Patients

**DOI:** 10.1155/2021/9590131

**Published:** 2021-09-17

**Authors:** Chi-Yung Cheng, I-Min Chiu, Wun-Huei Zeng, Chih-Min Tsai, Chun-Hung Richard Lin

**Affiliations:** ^1^Department of Emergency Medicine, Kaohsiung Chang Gung Memorial Hospital, Chang Gung University College of Medicine, Kaohsiung, Taiwan; ^2^Department of Computer Science and Engineering, National Sun Yat-sen University, Kaohsiung, Taiwan; ^3^Department of Pediatrics, Kaohsiung Chang Gung Memorial Hospital, Chang Gung University College of Medicine, Kaohsiung, Taiwan

## Abstract

**Background:**

Out-of-hospital cardiac arrest (OHCA) is a major health problem worldwide, and neurologic injury remains the leading cause of morbidity and mortality among survivors of OHCA. The purpose of this study was to investigate whether a machine learning algorithm could detect complex dependencies between clinical variables in emergency departments in OHCA survivors and perform reliable predictions of favorable neurologic outcomes.

**Methods:**

This study included adults (≥18 years of age) with a sustained return of spontaneous circulation after successful resuscitation from OHCA between 1 January 2004 and 31 December 2014. We applied three machine learning algorithms, including logistic regression (LR), support vector machine (SVM), and extreme gradient boosting (XGB). The primary outcome was a favorable neurological outcome at hospital discharge, defined as a Glasgow-Pittsburgh cerebral performance category of 1 to 2. The secondary outcome was a 30-day survival rate and survival-to-discharge rate.

**Results:**

The final analysis included 1071 participants from the study period. For neurologic outcome prediction, the area under the receiver operating curve (AUC) was 0.819, 0.771, and 0.956 in LR, SVM, and XGB, respectively. The sensitivity and specificity were 0.875 and 0.751 in LR, 0.687 and 0.793 in SVM, and 0.875 and 0.904 in XGB. The AUC was 0.766 and 0.732 in LR, 0.749 and 0.725 in SVM, and 0.866 and 0.831 in XGB, for survival-to-discharge and 30-day survival, respectively.

**Conclusions:**

Prognostic models trained with ML technique showed appropriate calibration and high discrimination for survival and neurologic outcome of OHCA without using prehospital data, with XGB exhibiting the best performance.

## 1. Introduction

Out-of-hospital cardiac arrest (OHCA) is a major public health problem worldwide, with an annual incidence of 50 to 100 per 100,000 in the general population [1]. OHCA has a high societal burden when compared to all other major causes of death, with an estimated 2.04 million years of potential life lost for men and 1.29 million years for women [2]. Despite advances in prehospital care, the prognosis for OHCA remains limited, with only 5.4%–20% [3–5] of patients surviving to hospital discharge. Neurologic injury remains the leading cause of morbidity and mortality among survivors of OHCA, because of inadequate cerebral perfusion during cardiac arrest or reperfusion injury that occurs in the early postresuscitation phase. The Pan Asian Resuscitation Outcomes Study (PAROS) Clinical Research Network demonstrated that the survival rate with proper neurological function was only 2.7% [5].

Many prehospital factors improve survival following OHCA, including witnessed cardiac arrest, bystander cardiopulmonary resuscitation (CPR), and initial heart rhythm [6–8]. The time from collapse to initiation CPR (no-flow interval) and the duration of CPR (low-flow interval) were also considered predictors of outcomes [9]. Severe scores were developed for predicting survival with proper neurological function at the time of ICU admission after OHCA. The OHCA score comprised five parameters, including the initial heart rhythm, no-flow interval, low-flow interval, serum creatinine, and arterial lactate [10]. The CAHP score stratified patients into three-level groups using seven variables, including age, initial heart rhythm, no-flow interval, low-flow interval, location of cardiac arrest, epinephrine dose, and arterial pH [11]. However, no-flow or low-flow intervals may be the result of inaccurate recall or recording during a highly stressful event. The updated Utstein template eliminated the necessity for recording the time of collapse, and thus, the duration of the no-flow interval could not be calculated [12].

In the past few years, machine learning (ML) techniques were used to influence clinical research and practice, such as prediction of sepsis through digital biomarker discovery [13], prediction of mortality for intensive care patients [14], and prediction of outcome in traumatic brain injury [15]. The ML algorithms outperform conventional triage tools and early warning scores in detecting patients at risk for cardiac arrest in emergency departments [16]. They can also accurately predict the need for critical care on information acquired during emergency medical services [17].

Previous studies have suggested that ML methods could predict neurologic and survival outcomes of OHCA patients [18–21]. Harford et al. found that an ML model can be used to support intervention decisions such as CPR or coronary angiography in OHCA patients [18]. However, only limited studies examined independent variables after patients arrived at the emergency department (ED). This study is aimed at investigating whether an ML algorithm could detect complex dependencies between clinical variables during ED in OHCA survivors and performing reliable predictions of the favorable neurological outcome.

## 2. Materials and Method

### 2.1. Study Setting and Variables

This was a retrospective study conducted from 1 January 2004 to 31 December 2014 in a tertiary medical center of southern Taiwan, which had 72,000 ED visits on average every year. The Ethics Committee of Chang Gung Memorial Hospital (No. 202001675B0) approved the study protocol. Because of the study's retrospective nature, informed consent from the subjects was not required.

The study included adults (≥18 years of age) who had a sustained return of spontaneous circulation (ROSC) after successful resuscitation from OHCA and were then admitted to ICU. The demographic characteristics, baseline comorbidities, and clinical variables were extracted from the ED electronic database. The underlying medical conditions included heart failure, cerebrovascular disease, peripheral vascular disease, diabetes mellitus, chronic obstructive pulmonary disease, chronic kidney disease, liver cirrhosis, malignancy, metastatic tumor, dementia, and moderate to severe Charlson comorbidity index (CCI) (CCI scored ≥3) [22]. Tentative diagnosis of cardiac arrest causes, such as hypothermia, hyperkalemia, acidosis (pH < 7.1), acute myocardial infarction (AMI), pulmonary embolism, tension pneumothorax, or intoxication, at the ED was recorded. Medication administration, including epinephrine, sodium bicarbonate, dopamine, norepinephrine, amiodarone, lidocaine, and calcium use or not, was collected. Intervention at ED included percutaneous coronary intervention and extracorporeal membrane oxygenation.

The primary outcome was a favorable neurological outcome at hospital discharge, defined as a Glasgow-Pittsburgh cerebral performance category (CPC) of 1 to 2. The favorable neurological outcome included patients with full recovery or those who can independently perform daily activities but may have a minor to moderate disability. However, CPC 3–5 was categorized as a poor functional outcome, which included patients dependent on others, in a coma or vegetative state, and who are dead [23, 24]. In this study, CPC scores were collected retrospectively using electronic medical records and physical examinations by a consensus of neurologists who were blinded to the study. The secondary outcome was the 30-day survival rate and survival-to-discharge rate.

### 2.2. Stepwise Feature Selection and ML Algorithms

To detect the model performance between features and subsequently select the best performing subset, all collected features were subjected to stepwise feature selection. The stepwise approach started with the evaluation of each individual feature based on forward feature selection and then checked for elimination. In each step, a variable was considered for addition to or subtraction from the set of explanatory variables based on mean accuracy.

We applied three ML algorithms including logistic regression (LR), support vector machine (SVM), and extreme gradient boosting (XGB). LR is a supervised classification algorithm. It transforms its output using a sigmoid function to return a probability value, which can then be mapped to two or more discrete classes. SVM belongs to the supervised learning technique for classification, increasingly used in many data mining and bioinformatics applications. SVM constructs a hyperplane based on the support vectors and maximizes the gap width between the two categories [25, 26]. XGB is a gradient boosted tree algorithm used for regression, binary and multiclass classification, and ranking problems. XGB is a robust and supervised learning algorithm capable of handling various data types, relationships, distributions, and hyperparameters that can be fine-tuned by users [27].

### 2.3. Outcome Prediction and Statistical Analysis

Categorical data are expressed as counts and proportions, and continuous data are expressed as means and standard deviations. The patients enrolled were randomly separated into the training set (90%) and test set (10%) for independent performance measurement of the model's generalizability. The training set was randomly divided into ten equal-sized groups for cross-validation during model development. We examined the area under the receiver operating characteristic curve (AUC) for performance measurement and plotted the receiver operating characteristic (ROC) curve using sensitivity against (1-specificity) [28]. We also compared positive predictive value (PPV) (true positives/(true positives+false positives)), sensitivity (true positives/(true positives+false negatives)), and specificity (true negatives/(true negatives+false positives)) between each prognostic model. The ML models were performed using Scikit-learn (version 0.22.2) with Python (version 3.8).

## 3. Experiment and Result

### 3.1. Dataset Description

For the study period, although there were 1076 patients, 1071 were included in our study for the final analysis. Five patients were excluded due to missing values. The mean age of the 1071 patients was 66.2 ± 16.8 years. The dataset included 596 (55.6%) males. There were 86 (8%) patients with favorable neurological outcomes after discharge. Furthermore, the dataset had 249 (23.2%) patients with 30-day survival and 216 (20.2%) patients survived to discharge. The other population characteristics were categorized and presented as underlying disease, laboratory data, medication, and intervention at ED. ED diagnosis is demonstrated in Table 1.

### 3.2. Feature Engineering

**Figure 1:**
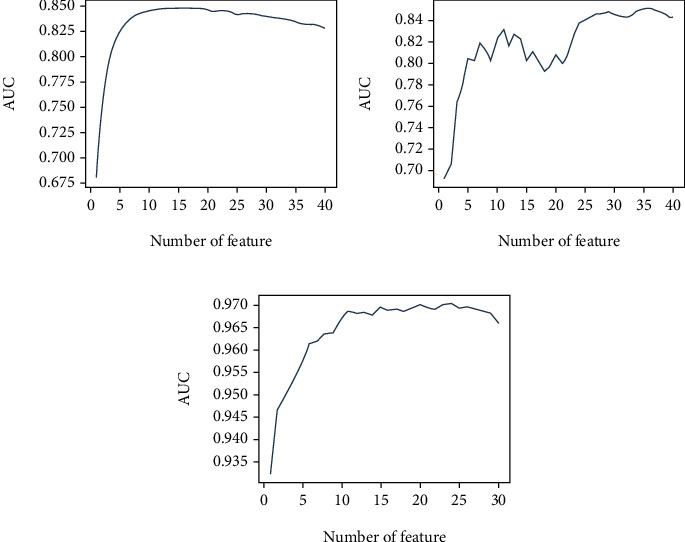
Forward stepwise feature selection of machine learning models based on AUC: (a) logistic regression; (b) support vector machine; (c) extreme gradient boosting.

All 42 variables were subjected to stepwise feature selection based on their individual importance and their effect on the mean accuracy to create the best performing subset prediction model.  Figure 1 depicts the results of stepwise feature selection for the three ML models.  Table 2 ranks the results of variables by importance. We used 10, 12, and 11 parameters for model training in the LR, SVM, and XGB algorithms, respectively. The parameters ranked by LR were PCI, DM, hemoglobin, troponin I, dementia, CCI, norepinephrine use, liver cirrhosis, hypokalemia, and tumor metastasis. For SVR, the features were troponin I, CCI, dementia, DKA, PCI, norepinephrine use, ECMO, pulmonary embolism, amiodarone use, pneumothorax, tumor metastasis, and acidosis. For XGB, the features were troponin I, epinephrine dose, heart failure, PCI, amiodarone use, calcium use, dementia, sodium bicarbonate use, band neutrophil, malignancy, and AMI.

### 3.3. Prediction

**Figure 2:**
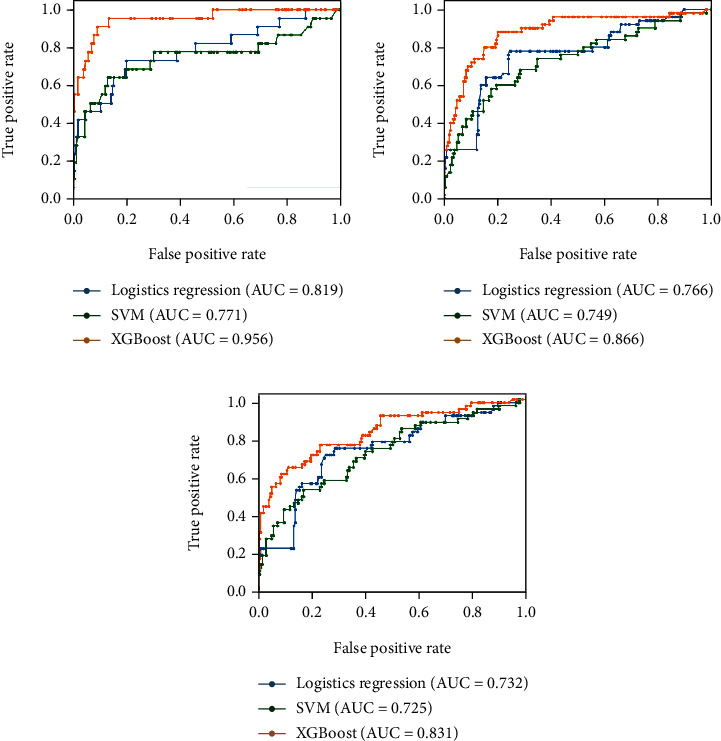
Receiver operating characteristic curve of three machine learning models: (a) favorable neurologic outcome; (b) survival-to-discharge; (c) 30-day survival.

Table 3 demonstrates the comparison of prediction ability for neurological outcomes between the three ML models. The AUC was 0.819, 0.771, and 0.956 in LR, SVM, and XGB, respectively. The sensitivity and specificity were 0.875 and 0.751 in LR, 0.687 and 0.793 in SVM, and 0.875 and 0.904 in XGB.  Table 4 presents the comparison of prediction ability for survival-to-discharge and 30-day survival. The AUC was 0.766 and 0.732 in LR, 0.749 and 0.725 in SVM, and 0.866 and 0.831 in XGB, for survival-to-discharge and 30-day survival, respectively.  Figure 2 depicts the ROC curve for the prediction performance of the three ML models.

## 4. Discussion

Using in-hospital data available within ED, we developed and validated different ML algorithms to stratify neurological outcomes after cardiac arrest. The AUC was 0.819, 0.771, and 0.956 in LR, SVM, and XGB, respectively. The sensitivity and specificity were 0.875 and 0.751 in LR, 0.687 and 0.793 in SVM, and 0.875 and 0.904 in XGB. The ML algorithm possessed suitable calibration and high discrimination in predicting favorable neurologic outcomes. For survival-to-discharge and 30-day survival prediction, the AUC was 0.766 and 0.732 in LR, 0.749 and 0.725 in SVM, and 0.866 and 0.831 in XGB, respectively. With acceptable outcome prediction ability, ML approaches are expected to improve clinician prognosis, earlier identification of outliers, information provision assistance, and physician-family communication.

In most of the current outcome prediction score and ML algorithms for OHCA, prehospital data are often implanted for predicting the variation in survival-to-discharge. The OHCA score, composed of five parameters, including no-flow and low-flow intervals, achieved an AUC of 0.82 in the development cohort and 0.88 in the validation cohort for neurological recovery outcome prediction [10]. Aschauer et al. discovered that using 21 variables, an LR model obtained an average AUC of 0.827 for survival probability, with key predictors being prehospital variables, such as the number of minutes to sustained restoration of spontaneous circulation and the first rhythm [29]. Another study cohort with 2639 patients, comparing several ML models (including decision tree, random forest (RF), *k*-nearest neighbors, XGB, light gradient boosting machine (GBM), and neural networks), stated that an embedded fully convolutional network model has the best average class sensitivity of 0.825 for neurological outcome prediction [18]. However, the above models required knowledge of the periods of time with circulatory no-flow and low-flow, limiting its use when prehospital data are unknown or recalled incorrectly. In our ML models, XGB exhibited the best performance with AUC of 0.956 for neurological outcome prediction, 0.866 for survival-to-discharge, and 0.831 for 30-day survival. The LR and XGB obtained a sensitivity of 0.875 for neurological outcome prediction. Without using prehospital data, the result of XGB was not inferior to previous models.

Nanayakkara et al.'s study from the Australian and New Zealand Intensive Care Society included 39,566 OHCA cases without prehospital data, and five ML approaches (GBM, SVM, RF, artificial neural network, and an ensemble) were compared for predicting mortality. With a combination of demographic, physiologic, and biochemical information, an ensemble and GBM could reach AUC of 0.87 (95% CI 0.86–0.88) for predicting in-hospital mortality [30]. Similarly, the AUC for XGB reached 0.866 and 0.831 for survival-to-discharge and 30-day survival prediction in our study, respectively. However, Nanayakkara et al.'s study did not discriminate survival from neurological outcomes. In contrast, we also found that XGB exhibited satisfactory performance in neurological outcome prediction. To our knowledge, this is the first study using ML models to predict functional neurological outcomes post-OHCA using only in-hospital variables.

We determined the order of importance among features and the best subsets of features using forward stepwise regression. A forward selection begins with no explanatory features and then adds features alternately, in each step, based on which feature is the most statistically significant, until all statistically significant features have been tested. The process selects explanatory variables for multiple regression models and develops the best combination of feature subsets. Although it has been criticized for misapplying single-step statistical tests to a multistep procedure, stepwise regression is efficient at narrowing down a long list of plausible explanatory variables to a manageable number of predictors [31]. Although different ML models disagreed on feature importance in our study, troponin I and PCI remained among the top five features among all three models. Because AMI is a common cause of OHCA, some studies have demonstrated that short-term outcomes after OHCA due to AMI can be better than that due to other causes of OHCA [32, 33].

Furthermore, our study faced several limitations. First, we did not include prehospital features in our study. Although many prehospital factors can improve survival following OHCA [6–8], the ML algorithms incorporate the result of mediation before the time when measurements were taken. In other words, the models had computed a vector component triggered by earlier intervention. Second, the dataset used in this study only included patients from a tertiary medical center in southern Taiwan. The findings of this study must be validated in a different region with a more ethnically diverse patient population.

## 5. Conclusion

Prognostic models trained using ML technique demonstrated appropriate calibration and high discrimination for survival and neurological outcome of OHCA, without the use of prehospital data, with XGB providing the best performance.

## Figures and Tables

**Table 1:** Characteristics of the patients at baseline.

Variables	All patients (*n* = 1071)
*Demographic characteristics*	
Age (years), mean ± SD	66.2 ± 16.8
Sex, male, *n* (%)	596 (55.6)
*Underlying medical conditions*, *n* (%)	
Heart failure	161 (15.0)
Cerebrovascular disease	248 (23.2)
Peripheral vascular disease	37 (3.5)
Diabetes mellitus	244 (22.8)
Chronic obstructive pulmonary disease	247 (23.1)
Chronic kidney disease	232 (21.7)
Liver cirrhosis	15 (1.4)
Malignancy	146 (13.6)
Tumor metastasis	23 (2.1)
Dementia	100 (9.3)
CCI scored ≥3	715 (61.8)
*Laboratory data,mean* ± *SD*	
White blood cell (1,000/*μ*L)	13.651 ± 7.4871
Segmented neutrophils (%)	53.05 ± 19.671
Band neutrophils (%)	2.36 ± 4.143
Hemoglobin (g/dL)	11.056 ± 2.9859
Creatinine (mg/dL)	2.570 ± 2.90
Alanine aminotransferase (ALT) (U/L)	248.97 ± 560.968
Na (mEq/L)	138.97 ± 7.935
K (mEq/L)	5.029 ± 1.588
Troponin I (ng/mL)	0.801 ± 5.364
pH	7.165 ± 0.226
*ED diagnosis*, *n* (%)	
Hypothermia	5 (0.5)
Hyperkalemia	216 (20.2)
Acidosis	722 (67.4)
Acute myocardial infarction	140 (13.1)
Pulmonary embolism	4 (0.4)
Tension pneumothorax	3 (0.3)
Toxin	30 (2.8)
Diabetes ketoacidosis	27 (2.5)
*Medication and intervention*	
Epinephrine use, *n* (%)	1050 (98.0)
Epinephrine dose, mean ± SD	5.35 ± 4.917
Sodium bicarbonate use, *n* (%)	690 (64.4)
Dopamine use, *n* (%)	655 (61.2)
Norepinephrine use, *n* (%)	212 (19.8)
Amiodarone use, *n* (%)	179 (16.7)
Lidocaine use, *n* (%)	38 (3.5)
Calcium use, *n* (%)	196 (18.3)
Defibrillation at ED, *n* (%)	93 (8.7)
PCI, *n* (%)	86 (8.0)
ECMO, *n* (%)	18 (1.7)
*Outcome*, *n* (%)	
CPC class 1 or 2	86 (8.0)
Survival-to-discharge	216 (20.2)
30-day survival	249 (23.2)

CCI: Charlson comorbidity index; PCI: percutaneous coronary intervention; ECMO: extracorporeal membrane oxygenation; CPC: cerebral performance category.

**Table 2:** Rank of parameter importance after stepwise parameter selection.

Rank	LR	SVM	XGB
1st	PCI	Troponin I	Troponin I
2nd	Diabetes mellitus	CCI	Total epinephrine dose
3rd	Hemoglobin	Dementia	Heart failure
4th	Troponin I	Diabetes ketoacidosis	PCI
5th	Dementia	PCI	Amiodarone use
6th	CCI	Norepinephrine use	Calcium use
7th	Norepinephrine use	ECMO	Dementia
8th	Liver cirrhosis	Pulmonary embolism	Sodium bicarbonate use
9th	Hypokalemia	Amiodarone use	Band neutrophil
10th	Tumor metastasis	Pneumothorax	Malignancy
11th		Tumor metastasis	Acute myocardial infarction
12th		Acidosis	

LR: logistic regression; SVM: support vector machine; XGB: extreme gradient boosting; PCI: percutaneous coronary intervention; CCI: Charlson comorbidity index; ECMO: extracorporeal membrane oxygenation.

**Table 3:** Area under the receiver operating curve, positive predictive value, sensitivity, and specificity between different machine learning models for neurologic outcome.

	LR	SVM	XGB
AUC	0.819 ± 0.017	0.771 ± 0.017	0.956 ± 0.003
PPV	0.229 ± 0.021	0.220 ± 0.044	0.437 ± 0.029
Sensitivity	0.875 ± 0.036	0.687 ± 0.005	0.875 ± 0.030
Specificity	0.751 ± 0.010	0.793 ± 0.004	0.904 ± 0.005

LR: logistic regression; SVM: support vector machine; XGB: extreme gradient boosting; AUC: area under the receiver operating curve; PPV: positive predictive value.

**Table 4:** Area under the receiver operating curve, positive predictive value, sensitivity, and specificity between different machine learning models for survival-to-discharge and 30-day survival.

	LR		SVM		XGB	
	Discharge	30 days	Discharge	30 days	Discharge	30 days
AUC	0.766 ± 0.020	0.732 ± 0.009	0.749 ± 0.013	0.725 ± 0.010	0.866 ± 0.006	0.831 ± 0.006
PPV	0.345 ± 0.016	0.354 ± 0.010	0.404 ± 0.018	0.368 ± 0.014	0.600 ± 0.029	0.564 ± 0.020
Sensitivity	0.780 ± 0.047	0.762 ± 0.019	0.720 ± 0.029	0.593 ± 0.021	0.840 ± 0.026	0.745 ± 0.018
Specificity	0.637 ± 0.012	0.579 ± 0.013	0.740 ± 0.009	0.692 ± 0.016	0.862 ± 0.005	0.825 ± 0.007

LR: logistic regression; SVM: support vector machine; XGB: extreme gradient boosting; AUC: area under the receiver operating curve; PPV: positive predictive value.

## Data Availability

The datasets used and/or analyzed during the current study are available from the corresponding authors on reasonable request.
